# Linear Hypopigmentation Following Subcutaneous Steroid Injection

**DOI:** 10.7759/cureus.63847

**Published:** 2024-07-04

**Authors:** Reema Tominna, Joseph Aleshaki, Ava Harrington, Morgan A Hatlovic, Humberto Gallego

**Affiliations:** 1 Osteopathic Medicine, Lake Erie College of Osteopathic Medicine-Bradenton, Bradenton, USA; 2 Dermatology, Sharp Rees-Stealy Medical Center, Santee, USA

**Keywords:** perilymphatic, linear, injection, hypopigmentation, fluoroscopy, corticosteroid

## Abstract

A 78-year-old woman presented to the dermatologist with linear hypopigmentation four weeks after a local corticosteroid injection. Corticosteroid injections are commonly used for various musculoskeletal conditions refractory to other conventional treatments. We discuss a case report of a patient with linear hypopigmentation in the perilymphatic distribution due to local corticosteroid injection for the treatment of carpal tunnel syndrome (CTS). Providers who routinely inject corticosteroids should discuss this rare complication prior to injections.

## Introduction

Corticosteroid injections are common practice in the treatment of various musculoskeletal conditions [1]. A rare complication of steroid injections is the occurrence of linear hypopigmentation. The exact mechanism of injury is unknown, but it is proposed that the corticosteroid from the injection can cause melanocyte destruction and loss of melanocyte function [2]. We discuss a case of linear hypopigmentation presenting after local corticosteroid injection for the treatment of carpal tunnel syndrome (CTS). This study aims to identify the importance of discussing an uncommon side effect of steroid injection.

## Case presentation

A 78-year-old African American woman initially presented to her primary care physician with a chief complaint of pain in the wrist. The patient was referred to musculoskeletal medicine for a fluoroscopy-guided steroid injection. Results revealed mild CTS, and the patient subsequently received a 0.25% bupivacaine and 40 mg triamcinolone injection administered to the dorsal left wrist. The patient noticed a small hypopigmented patch on the left dorsal hand two weeks later. After another two weeks, the patient presented to her primary care physician due to enlargement of the hypopigmented skin and was referred to dermatology.

**Figure 1:**
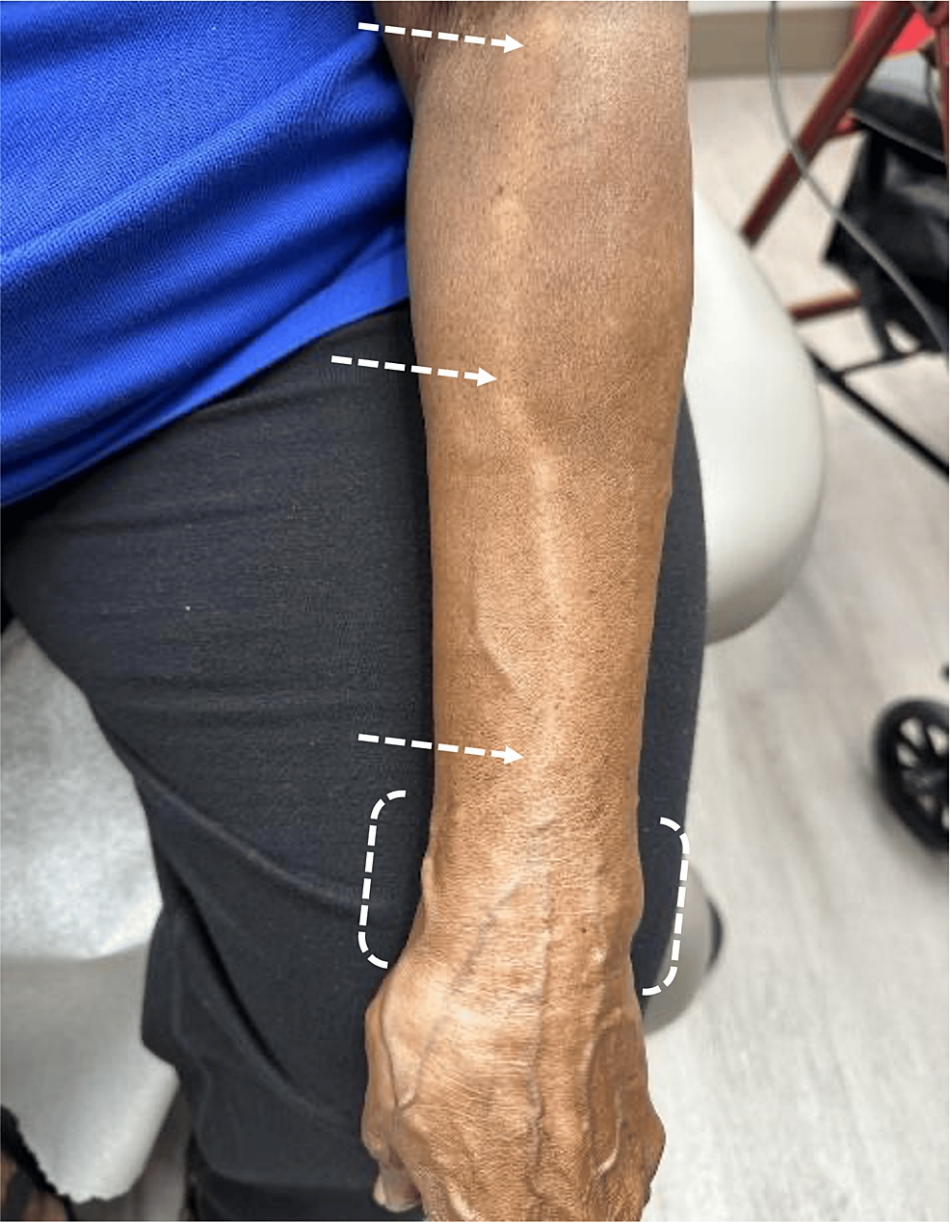
Linear hypopigmentation in the perilymphatic distribution on the left dorsal forearm after corticosteroid injection The dashed white arrows indicate the pattern of linear hypopigmentation. The dashed white brackets indicate the location of the steroid injection

**Figure 2:**
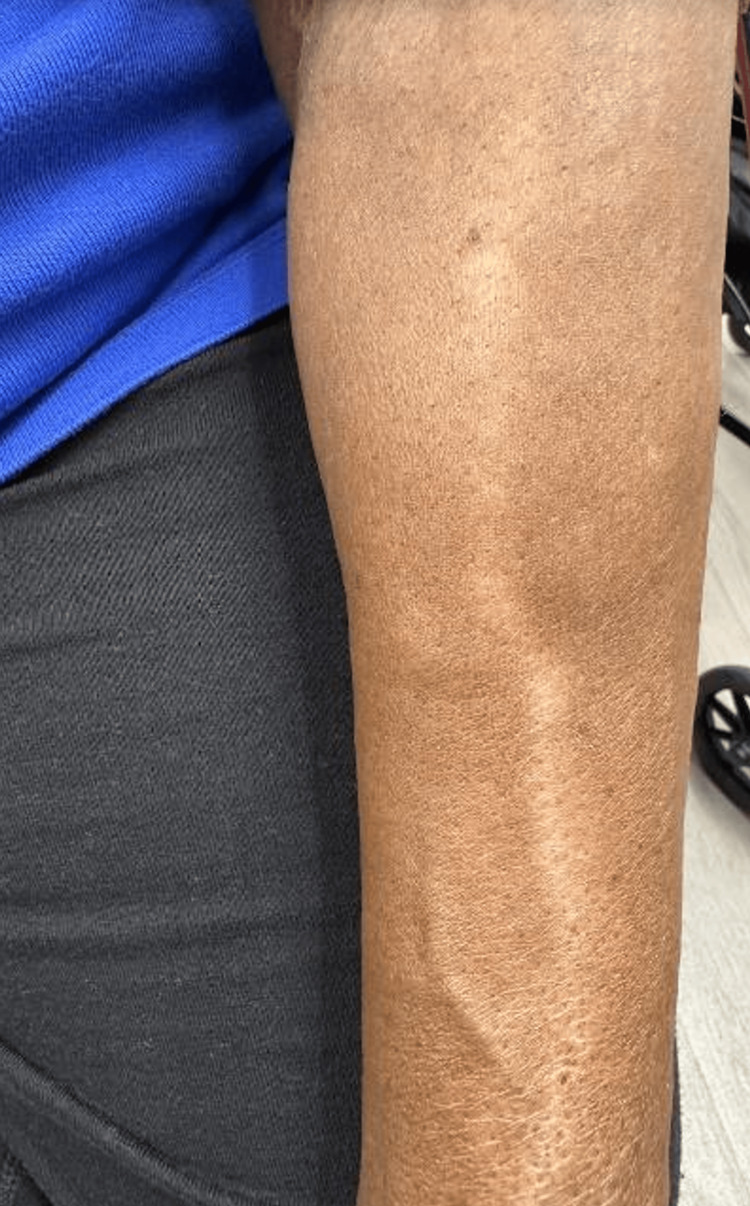
Hypopigmented lesion following a linear pattern proximally along the dorsal forearm and extending into the left antecubital fossa

The patient presented with a pattern of linear hypopigmentation originating near the site of the steroid injection on the left dorsal wrist (Figure 1, dashed white brackets). The cutaneous pattern travels linearly along the dorsal left forearm and extends proximally into the left antecubital fossa (Figure 1, dashed white arrows). The patient was otherwise asymptomatic and had no history of skin conditions or similar skin presentations in the past. Due to the clinical course of the hypopigmentation starting acutely after the steroid injection, without other identifiable triggers, the diagnosis of linear hypopigmentation in a perilymphatic distribution (Figures 1-2) following steroid injection was given. 

Management of linear hypopigmentation is minimal. It is possible that repigmentation can occur after about six months; however, patients should be aware that hypopigmentation may be permanent [3]. Physicians should discuss the cosmetic implications and provide reassurance that further systemic issues will not arise [3]. Our patient was informed about the risk that pigmentation could be permanent and to return to the clinic if any additional signs arose or if repigmentation occurred. However, our patient was lost to follow-up, possibly indicating that repigmentation did not occur. 

## Discussion

CTS is a common compression neuropathy of the median nerve most commonly at the flexor retinaculum and more prevalent in females [4]. After more conservative treatment options are tried, corticosteroid injection is a reasonable next step. Although the exact mechanism of corticosteroid injections to relieve CTS is unknown, many believe the anti-inflammatory component causes relief of CTS. Several studies have shown that a higher dose of methylprednisolone, triamcinolone, or hydrocortisone does not make a statistically significant difference, especially as a long-term treatment [1,5-7]. 

Corticosteroids are associated with various adverse effects including skin atrophy and hypopigmentation. While the exact mechanism of hypopigmentation is unknown, a proposed mechanism is the loss of melanocyte function instead of melanocyte destruction [8]. Additionally, a rare phenomenon is the occurrence of linear hypopigmentation induced by corticosteroid injections, which involves the lymphatic system as a conduit [9]. Importantly, topical steroids share these same adverse effects as injectable forms of steroids, commonly causing cutaneous side effects of skin atrophy in the form of striae and dyspigmentation [10]. 

The lymphatic system serves as a network of vessels responsible for the removal of proteins, macromolecules, and fluid from the interstitial spaces and tissues. When corticosteroids are administered through injection, they typically bind to plasma proteins such as albumin or corticosteroid-binding globulins. However, if the concentration of corticosteroids exceeds the binding capacity of these proteins, the excess corticosteroids are dispersed into the surrounding tissues and eliminated via the lymphatic system. This leads to the proposed hypothesis that hypopigmentation in the linear distribution may be due to the lymphatic uptake of the excess corticosteroid [2].

Some case reports have shown evidence of repigmentation after several months. Liang and McElroy reported repigmentation returning after six months. Triamcinolone showed an increased incidence of depigmentation [3].

In the case of our patient, a thorough history was conducted to identify the cause for her hypopigmentation. The patient admitted to receiving a steroid injection in the area of involvement four weeks prior to the appearance of the hypopigmentation. This allowed us to correlate our suspected etiology with the distribution of the hypopigmentation (Figures 1-2). This case study serves as a unique report of hypopigmentation along a lymphatic vessel following a corticosteroid injection for CTS.

## Conclusions

Perilymphatic hypopigmentation following subcutaneous corticosteroid injection is a rare complication. The proposed mechanism is that the volume of corticosteroid injected exceeds locally available binding globulins, causing free corticosteroid to disperse in surrounding tissues and subsequent uptake by lymphatic vessels. This leads to locally induced hypopigmentation and distribution along the lymphatic vessel. In the case of our patient, this occurred after subcutaneous corticosteroid injection for the treatment of CTS. Providers who routinely inject corticosteroids should discuss this rare complication prior to injection. Case reports discuss repigmentation after several months which may offer a resolution for those affected.
